# Naturally occurring beneficial bacteria *Vibrio alginolyticus* X-2 protects seaweed from bleaching disease

**DOI:** 10.1128/mbio.00065-23

**Published:** 2023-06-13

**Authors:** Mingyu Ma, Yingrui Zhuang, Lirong Chang, Luyang Xiao, Qin Lin, Qiying Qiu, Defu Chen, Suhelen Egan, Gaoge Wang

**Affiliations:** 1 College of Marine Life Science, Ocean University of China, Qingdao, China; 2 Institute of Evolution & Marine Biodiversity, Ocean University of China, Qingdao, China; 3 Weihai Changqing Ocean Science & Technology Co., Ltd, Rongcheng, China; 4 Fujian Lianjiang Guanwu Seafood Developing Product Co., Ltd, Guanwu, China; 5 Centre for Marine Science and Innovation & School of Biological, Earth and Environmental Sciences, The University of New South Wales, Sydney, New South Wales, Australia; University of Nebraska-Lincoln, Lincoln, Nebraska, USA

**Keywords:** *Saccharina japonica*, bleaching disease, beneficial bacteria, multi-omics, holobiont responses

## Abstract

Microbiome manipulation is gaining fresh attention as a way to mitigate diseases in aquaculture. The commercially farmed seaweed *Saccharina japonica* suffers from a bacterial-induced bleaching disease, which has major implications for the reliable supply of healthy sporelings. Here, we identify a beneficial bacterium, *Vibrio alginolyticus* X-2 that significantly reduces the risk of bleaching disease. By combining infection assays and multi-omic analyses, we provide evidence to suggest that the underlying protective mechanisms of *V. alginolyticus* X-2 involve maintaining epibacterial communities, increasing the gene expression of *S. japonica* related to immune and stress protection pathways, and stimulating betaine concentrations in *S. japonica* holobionts. Thus, *V. alginolyticus* X-2 can elicit a suite of microbial and host responses to mitigate the bleaching disease. Our study provides insights into disease control in farmed *S. japonica* through the application of beneficial bacteria.

**IMPORTANCE:**

Beneficial bacteria can elicit a suite of microbial and host responses to enhance the resistance to bleaching disease.

## INTRODUCTION

Microbiome manipulation using probiotics has been identified as an important approach to improve health and mitigate disease across a variety of host systems from human gut (1 - 3), to crops (4, 5) and aquaculture animals (6 - 10). Probiotic bacteria have been shown to function through indirect or direct antagonism of pathogenic bacteria (11), promoting a healthy microbiota (12) and/or boosting the host immune response (13). Plant growth–promoting microorganisms can protect *Arabidopsis thaliana* from root-derived filamentous eukaryotes and promote plant survival (14). In the case of marine organisms, for example, the addition of beneficial microorganisms to corals results in genetic and metabolic reprogramming of the host immune and stress responses that correlate with improved health outcomes and protection against bleaching disease (15, 16). Similar responses to probiotics have been observed in other aquatic animals such as fishes *Solea senegalensis* (17) and *Oncorhynchus mykiss* (18).

While the application of microbiota manipulation in seaweeds is still in its infancy, recent studies have identified beneficial bacteria that promote growth and/or improve disease resistance in some seaweeds (19). For example, specific bacteria found in the epimicrobiome of *Gracilaria conferta* prevent it from being damaged by tip necrosis (20). Similarly, the surface metabolites of the *Agarophyton vermiculophyllum* can attract protective bacteria that inhibit the settlement of pathogens, thereby reducing the risk of diseases (21). Moreover, a disease-protective bacterium, *Phaeobacter* sp. BS52, was identified in the wild red seaweed *Delisea pulchra* (22). The protective activity of *Phaeobacter* sp. BS52 was a result of mitigating and/or counteracting pathogen-induced bacterial dysbiosis, instead of direct pathogen inhibition. Despite the increasing awareness of the benefits of protective bacteria to their seaweed hosts, nothing is known about the molecular or metabolic response of seaweeds to these bacteria.

*Saccharina japonica* is one of the most important commercially farmed seaweeds globally. Similar to land crops, *S. japonica* suffers from various diseases at both the nursery and field cultivation stages (23, 24), such as hole-rotten disease (25, 26) and green rotten disease (27). These diseases cause 20%–50% of reduction in yields (23). Recently, a new form of bleaching disease resulting in frequent outbreaks during the late nursery stage of developing sporelings has been described (28). Bleached sporelings have white and rotted tips that eventually fall off. *Pseudoalteromonas piscicida* X-8 has been identified as an etiological agent of this bleaching disease (28) whereby pathogen exposure can result in a dysbiosis of the natural epiphytic bacterial community, leading to disease symptoms (29). Notably, during our initial isolation of the pathogen X-8, we obtained a second bacterial strain designated as X-2. Our preliminary studies showed that X-2 was antagonistic toward X-8 in antibacterial plate assays. However, it remains unknown whether X-2 can reduce the risk of X-8-induced bleaching disease *in vivo* and whether exposure to either strain elicits a specific host response.

In this study, by combining *in vivo* infection assays and multi-omics approaches, we aim first to characterize and confirm strain X-2 as a beneficial bacterium against bleaching disease in *S. japonica*, and second to explore the underlying mechanisms of disease protection at the level of (i) epimicrobiota changes, (ii) host gene expression, and (iii) metabolic responses. Our study not only provides new insights into the mechanisms of the interactions between the beneficial bacteria, epimicrobiome members, and the hosts in the area of commercially farmed seaweeds but will also contribute to the development of beneficial bacteria as a novel strategy to control disease outbreaks in *S. japonica* cultivation.

## RESULTS

### Identification of a beneficial bacterium for *S. japonica*

**Fig 1:**
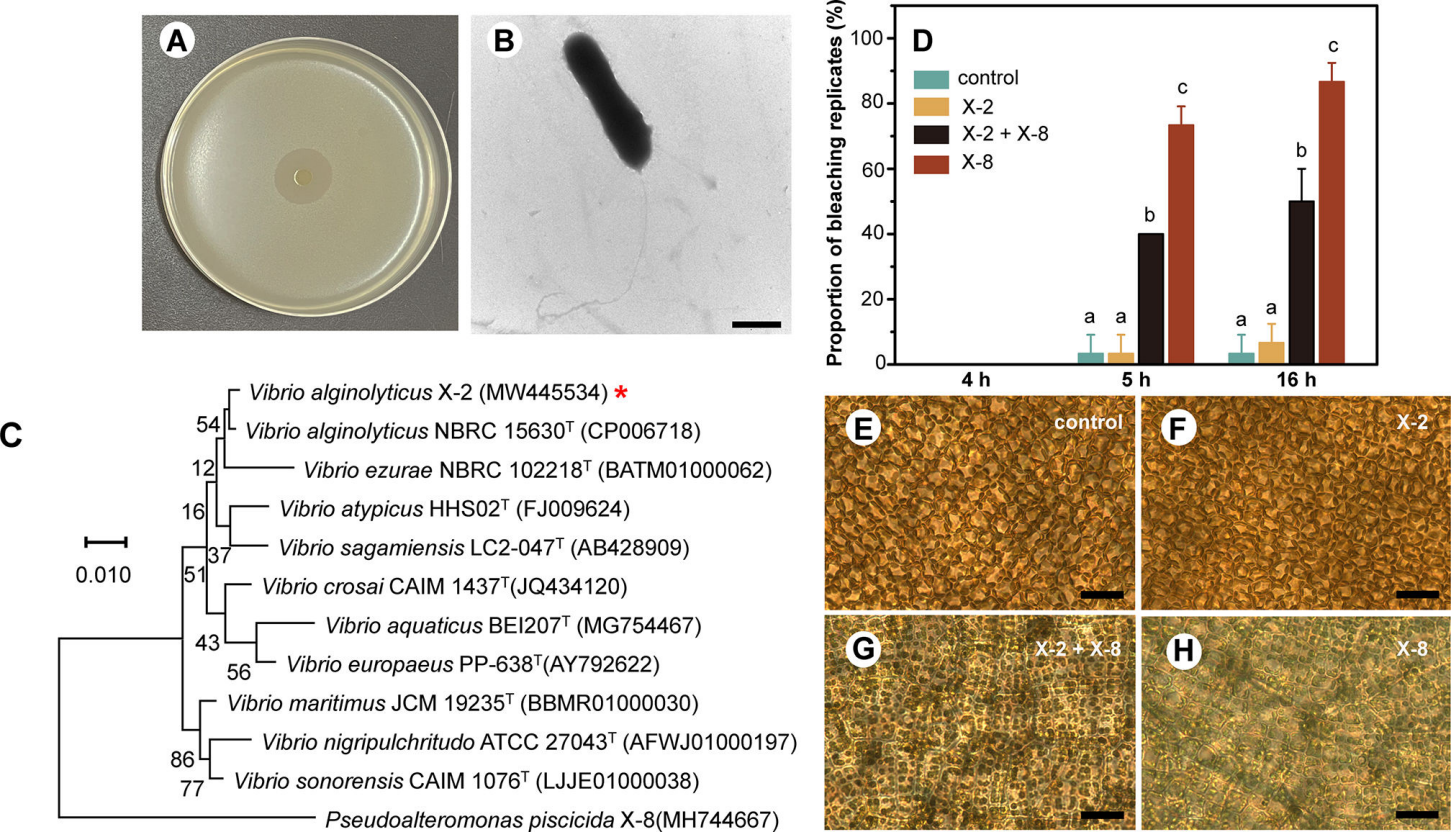
Identification of the beneficial bacterium strain X-2. (**A**) Antagonistic activity of X-2 against the pathogenic bacteria X-8, the zone of growth inhibition was 19 mm. (**B**) Morphological observation of strain X-2 by negative-staining TEM. (**C**) Maximum likelihood phylogenetic tree based on 16S rRNA gene sequences. *Pseudoalteromonas piscicida* X-8 was used as an outgroup. The position of strain X-2 is highlighted with a red asterisk. The accession numbers of different species are exhibited in parentheses. Bootstrap values for 1,000 replicates are shown at the branch nodes. (**D**) The proportion of *S. japonica* bleaching replicates. Statistical differences (*P* < 0.05) between treatments are shown by different lowercase letters. (**E–H**) Microscopic observations of *S. japonica* tissues inoculated with sterile seawater (control), X-2, X-2 +X-8, and X-8, respectively. Bars: A: 10 mm; B: 1 µm; E, F, G, and H: 50 µm.

Strain X-2 was isolated from diseased *S. japonica*, at the same time as the pathogenic strain X-8. Antagonistic bioassays demonstrated that strain X-2 was able to inhibit the growth of X-8 (Fig. 1A). Negative-staining TEM showed that strain X-2 is rod shaped with a single polar flagellum (Fig. 1B). The full-length 16S rRNA gene sequence (1,553 bp) of strain X-2 (accession no. MW445534 at https://www.ncbi.nlm.nih.gov/nuccore/MW445534) and subsequent phylogenetic analysis indicate that X-2 belongs to the species *V. alginolyticus*, with the highest gene homology to strain NBRC 15630^T^ (99.66%) (Fig. 1C). We also compared the whole-genome sequence of strain X-2 (NCBI BioProject: PRJNA810890 at https://www.ncbi.nlm.nih.gov/bioproject/PRJNA810890) with *V. alginolyticus* NBRC 15630^T^ and found an 98.35% ANI (average nucleotide identity) further supporting the assignment of X-2 to *V. alginolyticus*. Accordingly, we designated X-2 as *V. alginolyticus* X-2.

We then tested for the ability of strain X-2 to protect *S. japonica* from X-8-induced bleaching *in vivo*. Inoculation of *S. japonica* with the pathogen alone resulted in the onset of bleaching disease after 5 hours, with the proportion of bleached individuals in this treatment increasing up to 16-hour post-inoculation (Fig. 1D). In contrast, inoculation with strain X-2 had no significant effect on algal health compared to uninoculated controls (*P* > 0.05; Fig. 1D). However, co-inoculation with X-2 and X-8 (X-2:X-8 = 1:10) significantly reduced the proportion of the bleaching replicates compared to the pathogen (i.e., X-8) treatment alone (*P* < 0.001) (Fig. 1D), which is in line with the antagonistic assays. These results were supported by microscopic observations (Fig. 1E through H), highlighting inoculation with X-2 protects against morphological/cellular damage.

### Co-culture with the beneficial bacterium X-2 results in changes in the epibacterial community of *S. japonica*

The epibacterial communities of *S. japonica* in different treatments were analyzed to evaluate the effect of the beneficial bacteria after 16 hours (the time point where the proportion of bleached individuals significantly increased) of exposure. After sequencing and quality filtering, a total of 3,745,801 sequences and 5,486 ASVs were obtained (Table S1 at https://doi.org/10.6084/m9.figshare.22337065). Rarefaction curves and Good’s coverage estimates (>99%) indicated that the sequencing captured the majority of the bacterial diversity in the samples (Fig. S1 at https://doi.org/10.6084/m9.figshare.22337065).

**Fig 2:**
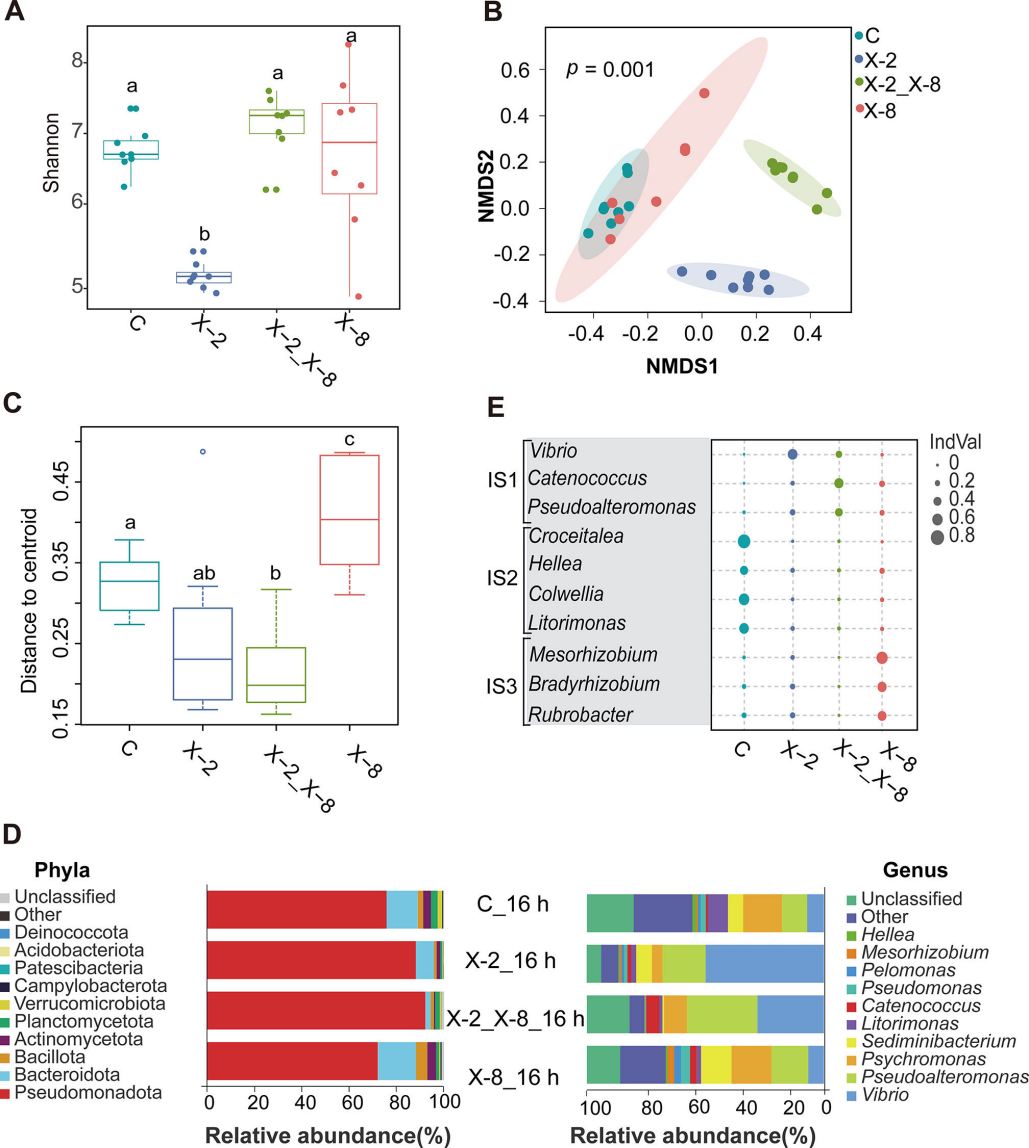
The shift of epibacterial community associated with *S. japonica* 16-hour post-inoculation with X-2, X-2 + X-8, and X-8. (**A**) Alpha-diversity (Shannon index) of the epibacterial community associated with *S. japonica* after treatment with control, X-2, X-2 + X-8, and X-8 at 16 hours. Statistical differences (*P* < 0.05) between treatments are shown by different lowercase letters. (**B**) NMDS plots of *S. japonica* epibacterial community structure (Bray–Curtis dissimilarity) in different treatments. (**C**) Dispersions analysis of epibacterial community structure using permutational analysis of multivariate dispersions (PERMDISP). (**D**) Epibacterial community composition at the phylum and genus levels in different treatments. (**E**) Genera with relative abundances >1% and IndVal significantly different in different treatments. IS represents genera with significantly higher IndVal in X-2 inoculated and X-2 + X-8 co-inoculated (IS1), control (C, IS2), and X-8 inoculated treatments (IS3), respectively (*P* < 0.05).

To confirm the presence of the inoculated bacteria, the 16S rRNA gene sequences of strains X-2 and X-8 were searched against the ASVs in each sample. The sequences of ASV000007 and ASV000001 matched that of X-2 and X-8, respectively. Subsequent bacterial community analyses were conducted after subtracting the X-2 and X-8 reads from the data set. We found that inoculation with X-2 had no significant effect on richness (Fig. S2) but significantly reduced the diversity of the epibacterial community associated with *S. japonica* compared to the controls and either co-inoculation of X-2 + X-8 or X-8 alone (*P* < 0.001; Fig. 2A). In addition, the epibacterial community structure of the different treatments showed significant separation (PERMANOVA; *P* = 0.001) and pairwise comparisons between treatments were also significantly different (Fig. 2B; Table S2). However, a test of dispersion showed that treatment with X-8 alone resulted in epibacterial communities that were more dispersed than the controls or communities in the presence of the beneficial strain X-2 (PERMDISP, *P* = 0.001; Fig. 2C; Table S3).

Members of Pseudomonadota and Bacteroidota were the most abundant phyla across all treatments, with distinct differences between treatments at the genus level. For example, the most abundant genera in the control included *Psychromonas* (16.3%) and *Pseudoalteromonas* (10.7%). *Vibrio* (49.9%) and *Pseudoalteromonas* (18.2%) were the most abundant genera in the X-2 treatment. *Vibrio* (28.1%) and *Pseudoalteromonas* (29.7%) were the most abundant genera in the X-2 + X-8 co-inoculated treatment, and *Psychromonas* (16.8%) and *Pseudoalteromonas* (15.6%) were the most abundant genera in the X-8 alone treatment (Fig. 2D). We further identified a total of 12 genera with a relative abundance > 1% that were indicative of a particular treatment (Fig. 2E), including *Vibrio* that was indicative of treatments with X-2 and *Mesorhizobium*, *Bradyrhizobium*, and *Rubrobacter* that were indicative of treatment with X-8 alone (Fig. 2E). Overall, inoculation with the beneficial bacterium X-2 resulted in the shift of epibacterial community associated with *S. japonica,* compared to the X-8 alone treatment, which may play a role in mitigating bleaching disease.

### Beneficial bacterium X-2 elicits changes in the transcriptome of *S. japonica*

**Fig 3:**
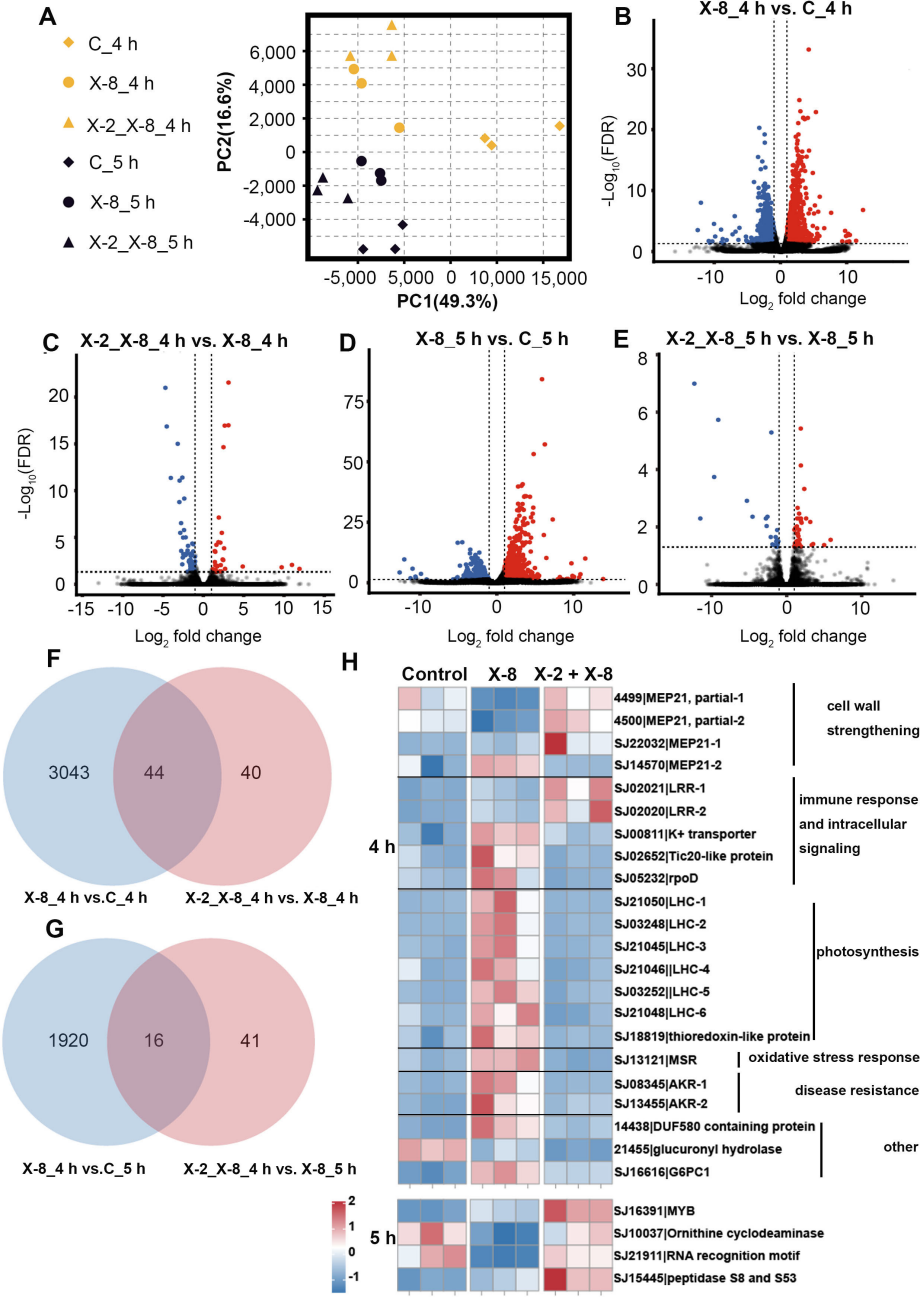
Analysis of differentially expressed genes (DEGs) between X-2 + X-8 co-inoculated and X-8 inoculated treatments. (**A**) Principal component analysis (PCA) plots of *S. japonica* transcripts obtained by RNA-seq after 4 and 5 hours of exposure to both X-2 + X-8 and X-8 treatments. (**B**) Volcano plot of DEGs between X-8 and control (seawater only) treatments after 4 hours of exposure. Each point in the volcano plot represents a gene, the red points represent the up-regulated genes in X-8 treatment, the blue points represent the down-regulated genes, and the black points represent the genes with no significant changes. (**C**) Volcano plot of DEGs between X-2 + X-8 and X-8 treatments after 4 hours of exposure. Each point in the volcano plot represents a gene, the red points represent the up-regulated genes in X-2 + X-8 co-inoculated treatment, the blue points represent the down-regulated genes, and the black points represent the genes with no significant changes. (**D**) Volcano plot of DEGs between X-8 and control treatments after 5 hours of exposure. Each point in the volcano plot represents a gene, the red points represent the up-regulated genes in X-8 treatment, the blue points represent the down-regulated genes, and the black points represent the genes with no significant changes. (**E**) Volcano plot of DEGs between X-2 + X-8 and X-8 treatments after 5 hours of exposure. Each point in the volcano plot represents a gene, the red points represent the up-regulated genes in X-2 + X-8 co-inoculated treatment, the blue points represent the down-regulated genes, and the black points represent the genes with no significant changes. (**F**) Venn diagram of DEGs in *S. japonica* between treatments after 4 hours of exposure. (**G**) Venn diagram of DEGs in *S. japonica* between treatments after 5 hours of exposure. (**H**) DEGs can be annotated to specific proteins between treatments after 4 and 5 hours of exposure, respectively. The names of DEGs and their related pathways or functions are listed in the right. AKR, ankyrin repeat–containing protein; FDR, false discovery rate; G6PC1, alpha-(1, 6)-fucosyltransferase; LHC, light harvesting complex protein; LRR, leucine-rich repeat carbohydrate-binding protein; MEP21, mannuronan C-5-epimerases; MSR, methionine-R-sulfoxide reductase.

We used an RNA-seq approach to assess the host response to X-2 + X-8 and X-8 treatments after 4 hours (before the onset of bleaching symptom) and 5 hours (the onset of bleaching symptom) of exposure (Table S4). PCA and PERMAOVA showed overall differences between treatments (*P* = 0.001) and distinct separation over time (*P* = 0.001; Fig. 3A; Table S5).

A total of 3,087 DEGs (1,723 up-regulated and 1,364 down-regulated, *P* < 0.05, FC > 2 or < 0.5; Fig. 3B; Table S6) were detected in X-8 versus control (seawater only) treatment, and 84 DEGs (32 up-regulated and 52 down-regulated; Fig. 3C; Table S6) were detected in X-2 + X-8 versus X-8 treatment after 4 hours exposure. In contrast, fewer DEGs were detected in response to X-8 versus control treatment (total 1,936 DEGs including 946 up-regulated and 990 down-regulated; Fig. 3D; Table S6) and X-2 + X-8 versus X-8 treatment (total 57 DEGs including 37 up-regulated and 20 down-regulated; Fig. 3E; Table S6) after 5 hours exposure.

To determine how beneficial bacteria influence the transcriptome response of *S. japonica* to the pathogen, we focus on the common DEGs between X-8 versus control treatment and X-2 + X-8 versus X-8 treatment. After 4 hours of exposure, a total of 44 common DEGs of the two comparison groups were detected, of which 22 genes were likely pseudogenes or encoded unnamed proteins and 22 genes can be annotated to specific proteins (Fig. 3F and H; Table S6). Many of the common DEGs were involved in cell wall components (mannuronan C-5-epimerases, MEP21). Most of the *MEP21*s were down-regulated in X-8 compared to the control treatment, while they were up-regulated in X-2 + X-8 compared to the X-8 treatment. In addition, two genes encoding leucine-rich repeat carbohydrate-binding proteins (LRR) associated with immune response and intracellular signaling were up-regulated in both comparison groups. However, a large number of genes related to photosynthesis and oxidative stress response and two genes encoding ankyrin repeat–containing protein (*AKR*) were up-regulated in X-8 compared to control treatment and were down-regulated in X-2 + X-8 compared to X-8 treatment. In comparison after 5 hours of exposure, 16 DEGs were common between the two comparison groups, of which 12 genes were likely pseudogenes or encoded proteins of unknown function, with the remaining 4 genes annotated to specific functions (Fig. 3G and H; Table S6). Similar to 4 hours post-inoculation, the genes associated with immune response and intracellular signaling were up-regulated in both comparison groups.

To investigate if the changes in the transcriptome of *S. japonica* were in response to strain X-2 or were a result of mitigating the effect of X-8, we analyzed the differential expression of the common DEGs mentioned above in X-2 compared to the control treatment. We found that most of the common DEGs were not significantly different between X-2 and the control (Table S7), suggesting that X-2 plays a protective role mainly through mitigating X-8-induced changes in *S. japonica* transcriptome rather than through a direct action of X-2.

To validate the RNA-seq data, we selected five DEGs in different pathways to perform quantitative RT-PCR (qRT-PCR). The qRT-PCR results showed a significant correlation with RNA-seq result (*R*^2^ = 0.815, *P* < 0.01; Fig. S4), supporting the DEG analysis.

**Fig 4:**
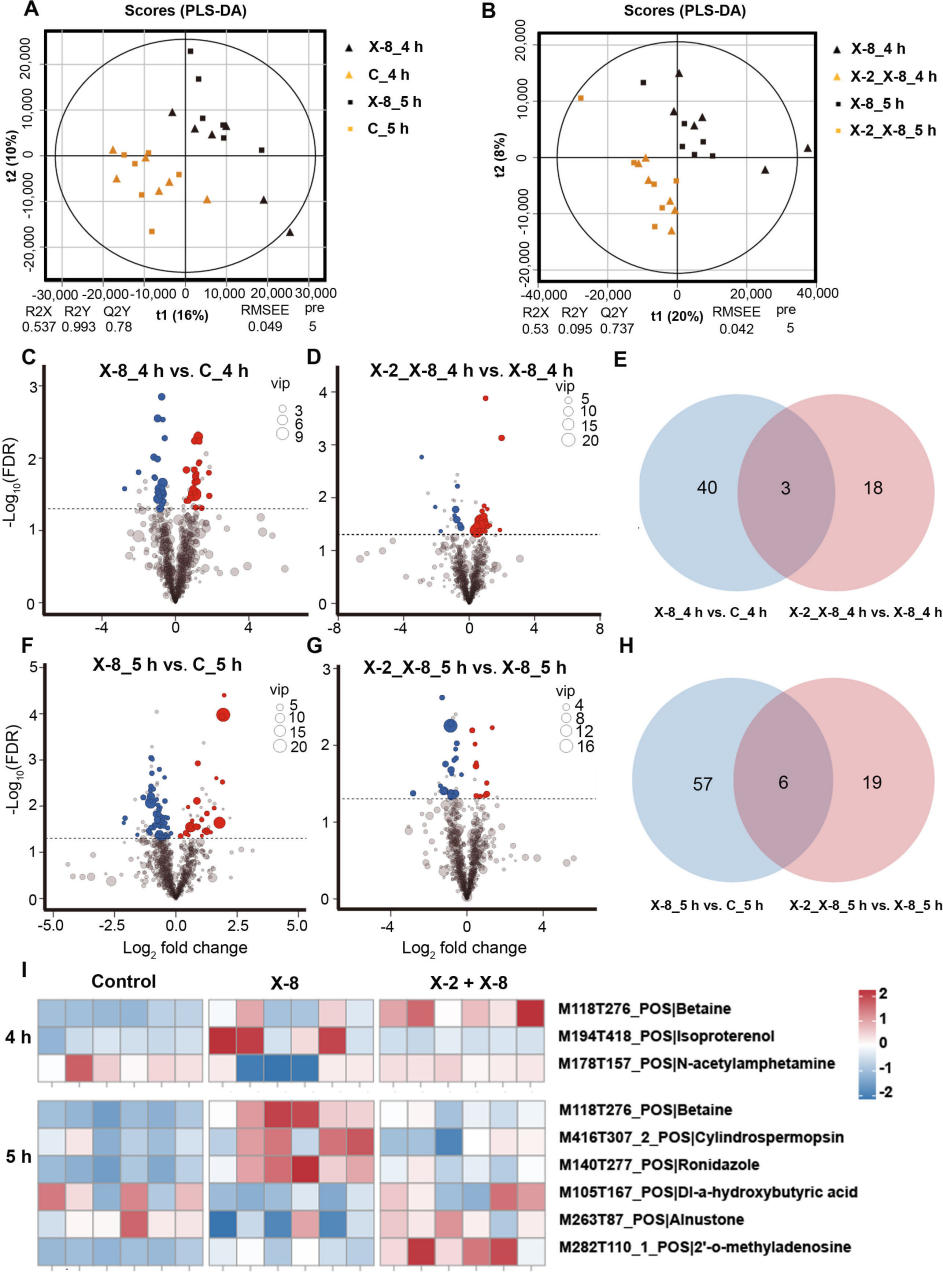
Analysis of DMs between X-2 + X-8 co-inoculated and X-8 inoculated treatments. (**A**) Partial least squares discriminant analysis (PLS-DA) of *S. japonica* holobiont metabolites, measured by both ESI+ and ESI− modes, after 4 and 5 hours of exposure to control and X-8 treatments. (**B**) PLS-DA of *S. japonica* holobiont metabolites, measured by both ESI+ and ESI− modes, after 4 and 5 hours of exposure to X-2 + X-8 and X-8 treatments. (**C**) Volcano plot of differential metabolites (DMs) between control and X-8 treatments after 4 hours of exposure. Each point in the volcano plot represents a metabolite, the red points represent the up-regulated metabolites in X-8 treatment, the blue points represent the down-regulated metabolites, and the black points represent the metabolites with no significant changes. (**D**) Volcano plot of DMs between X-2 + X-8 and X-8 treatments after 4 hours of exposure. Each point in the volcano plot represents a metabolite, the red points represent the up-regulated metabolites in X-2 + X-8 co-inoculated treatment, the blue points represent the down-regulated metabolites, and the black points represent the metabolites with no significant changes. (**E**) Venn diagram of significantly DMs in *S. japonica* between treatments after 4 hours of exposure. (**F**) Volcano plot of DMs between control and X-8 treatments after 5 hours of exposure. Each point in the volcano plot represents a metabolite, the red points represent the up-regulated metabolites in X-8 treatment, the blue points represent the down-regulated metabolites, and the black points represent the metabolites with no significant changes. (**G**) Volcano plot of DMs between X-2 + X-8 and X-8 treatments after 5 hours of exposure. Each point in the volcano plot represents a metabolite, the red points represent the up-regulated metabolites in X-2 + X-8 co-inoculated treatment, the blue points represent the down-regulated metabolites, and the black points represent the metabolites with no significant changes. (**H**) Venn diagram of significant DMs in *S. japonica* between treatments after 5 hours of exposure. (**I**) DMs can be annotated to specific proteins between treatments after 4 and 5 hours of exposure, respectively. The names of DMs and their related pathways or functions are listed on the right. FDR, false discovery rate.

### Beneficial bacterium X-2 elicits changes in the metabolome of *S. japonica* holobiont

We further explored the metabolic response of *S. japonica* holobionts using UHPLC-MS/MS after 4 and 5 hours of exposure to X-2 + X-8 or X-8. Metabolites from control, X-8 inoculated, and X-2 + X-8 co-inoculated treatments were separated according to the PLS-DA, indicating that bacterial inoculation resulted in changes in the metabolome of *S. japonica* holobionts (Fig. 4A and B). We focused on the DMs that could be annotated with Mass Spectra Second Class name (MS2_name).

After 4 hours of exposure, there were 43 and 21 DMs (*P* < 0.05, FC > 1.5 or < 0.667, and VIP > 1.0; Fig. 4C and D; Table S8) in X-8 versus control treatment and X-2 + X-8 versus X-8 treatment, respectively. Similarly, a total of 63 and 25 DMs (*P* < 0.05, FC > 1.5 or < 0.667, and VIP > 1.0; Fig. 4F and G; Table S8) were detected in response to X-8 relative to the control treatment and X-2 + X-8 relative to X-8 treatment, respectively, after 5 hours of exposure.

We also detected the common DMs between X-8 relative to the control treatment and X-2 + X-8 relative to X-8 treatment to determine how beneficial bacteria influence the *S. japonica* holobiont metabolome response to the pathogen. After 4 hours of exposure, a total of three common DMs between two comparison groups were detected (Fig. 4E). Of these DMs, betaine was more abundant in the X-2 + X-8 treatment (Fig. 4I). However, after 5 hours, six common DMs between two comparison groups were detected (Fig. 4H). In contrast to the observed changes in DMs after 4 hours, we found that metabolites such as betaine were significantly less abundant in the X-2 + X-8 co-inoculated treatment than X-8 alone at 5 hours (Fig. 4I). Moreover, similar to the transcriptome, most of the common DMs were not significantly different between X-2 versus control (Table S9). This further supported that X-2 played the protective role mainly through mitigating X-8-induced metabolome changes in *S. japonica* rather than through X-2 itself.

To further elucidate the relationship between DMs, the *S. japonica* epibacterial community, and transcriptome changes, we performed the correlation analyses between top 20 ASVs, the above mentioned common DEGs and DMs with annotation using Spearman correlation analysis. We observed significant correlations between the majority of DEGs and DMs (Fig. S5 and S8) at both 4- and 5-hour time points. In addition, the analysis showed that *Vibrio* (including ASV000006, 10, 14, 15, 16, and 19), *Psychromonas* (ASV000008), *Pseudomonas* (ASV000012), *Pseudoalteromonas* (ASV000013), *Psychromonas* (ASV000017), *Pelomonas* (ASV000018), and *Catenococcus* (ASV000024) were significantly correlated with most DEGs and DMs (Fig. S6, S7, S9, and S10).

## DISCUSSION

Microbiome manipulation through probiotics can mitigate or rebalance pathogen-induced microbiome dysbiosis, eventually promoting host fitness (3 - 5). Nevertheless, few studies have investigated seaweed probiotics (20 - 30 - 30) and little is known about their mechanisms of action. Here, we identified a beneficial bacterium, *V. alginolyticus* X-2, that can mitigate the occurrence of bleaching disease in commercially farmed *S. japonica*. Our results showed that the addition of strain X-2 results in the restructuring of epibacterial communities, the alteration of host gene expression (up-regulated genes involved in immune and stress protection pathways, down-regulated genes involved in energy synthesis of the photosynthetic pathway), and metabolism (biosynthesis of betaine).

Disease mitigation through the addition of beneficial microbiota has recently been documented in native populations of the red seaweed *D. pulchra* (22). Co-inoculation of the protective bacterium *Phaeobacter* sp. BS52 (BS52) with the pathogen *Aquimarina* sp. AD1 (AD1) in *D. pulchra* reduced the incidence of mid-thallus bleaching. It was concluded that the protective activity of BS52 was a result of mitigating bacterial dysbiosis, instead of directly inhibiting the growth of the pathogen AD1. Similarly, we found that X-2 is also able to mitigate the pathogen-induced epimicrobiota dysbiosis in *S. japonica*. Compared to those inoculated with X-8 alone (Fig. 2B), the structure of epibacterial community was less dispersed among the eight replicates of the X-2 + X-8 co-inoculation treatment. We speculate that X-2 influences other members of the epimicrobiota of *S. japonica* to some extent, which contributes to the stability of epibacterial communities. We found that X-2 can increase the relative abundance of ASVs corresponding to *Vibrio*. Some members of the *Vibrio* have been reported as a source of compounds with antibacterial activity (31) and had antagonistic activities against bacterial pathogens such as *Micrococcus luteus* and *Pseudomonas putida* of brown alga *Splachnidium rugosum* (32). It is likely that *Vibrio* plays a similar protective role for *S. japonica* and that the treatment with X-2 increases the relative abundance of *Vibrio* and indirectly maintains the stability of epibacterial communities to reduce the onset of bleaching disease in *S. japonica*.

However, unlike the antagonistic activity test, after analyzing the relative abundances of X-2 and X-8 based on 16S rRNA gene amplicon sequencing, we did not find statistical support for a decreased relative abundance of X-8 in the X-2 and X-8 co-inoculation treatment in infection assays (Fig. S3). Similar results have also been reported between the beneficial bacterium BS52 and the pathogen AD1 in red seaweed *D. pulchra* (22). BS52 inhibited the growth of AD1 identified by an antagonistic bioassay, and co-inoculation with BS52 and AD1 can decrease the bleaching proportion of *D. pulchra*. Analysis of 16S rRNA gene amplicon sequencing data indicated that there was no negative correlation between the relative abundances of BS52 and AD1, and the protection of BS52 was supposed to mitigate the pathogen-induced changes in the epibacterial community, not directly inhibiting the growth of the pathogen AD1. It is possible that, as has been proposed for the relationship between the beneficial bacterium BS52 and the pathogen AD1 in *D. pulchra*, X-2 may protect *S. japonica* against bleaching disease by inhibiting virulence of the X-8 instead of direct growth inhibition. Moreover, X-2 may help to maintain the stability of the epimicrobiota associated with *S. japonica* and to elicit defense responses in the transcriptome and metabolome of the *S. japonica* holobiont to play a protective role.

Probiotics can confer benefits to their hosts not only by manipulating the microbial community but also by enhancing the host’s response to disease (33, 34). For example, it has been reported that probiotics can promote the antioxidative status and stimulate immune signaling pathways against infectious diseases in marine animals, such as red sea bream (35), white shrimp (36), and sea bass (37). Similarly, we observed significant differences in the expression of genes involved in immune defense and antioxidant response between X-2 + X-8 co-inoculated and X-8 inoculated treatments. Genes encoding LRR, which play a regulatory role in innate immunity in plants (38), were induced after 4 hours of exposure to X-2 + X-8 treatment. It is possible that X-2 contributed to the enhanced immune responses. Moreover, X-2 also influenced cell-wall components and photosynthesis to increase the resistance of *S. japonica* to pathogen infection. Compared to the control treatment, several homologous genes, encoding MEP21, were down-regulated after 4 hours of exposure to X-8. But they were up-regulated in response to X-2 + X-8 relative to X-8 treatment. MEP21s play a central role in catalyzing the final step in the biosynthesis of alginate, a major cell-wall constituent of brown algae, resulting in cell-wall strengthening (39, 40). The cell wall is the first line of defense against pathogen infection, suggesting that X-8 can weaken the cell wall of *S. japonica,* and the one way in which X-2 acts to mitigate disease is via cell-wall strengthening. In contrast, the majority of DEG-encoded components of the photosynthetic pathway were down-regulated in response to co-inoculation with X-2 + X-8 compared to treatment with X-8 alone. A previous study in *Arabidopsis thaliana* indicated that the reduced expression of photosynthesis-related genes enhances the host defense response because the slow turnover of photosynthesis-related proteins allows cellular energy to be reallocated to defense needs (41). An increased defense capacity of *S. japonica* against pathogens induced by X-2 is further supported by the down-regulation of several homologous genes, encoding AKR in X-2 + X-8 co-inoculated treatment. It has been reported that the reduced expression of the *Arabidopsis AKR2* significantly increased the resistance to pathogen infection (42). Similarly, the repression of *AKR*s could also enhance the defense mechanisms of *S. japonica*. In addition, methionine sulfoxide reductase plays a crucial role in the repair of oxidatively damaged methionine residues in proteins (13). Therefore, while further work is required, the repression of this gene suggests that *S. japonica* has a higher level of protection against oxidative damage after inoculation with X-2.

After 5 hours of exposure, fewer common DEGs of X-8 versus control and X-2 + X-8 versus X-8 were detected than after 4 hours. According to the infection assays in this study, the onset of bleaching disease symptoms occurred at 5 hours. Therefore, we suggest that X-2 is likely to be more effective as a protective agent before the onset of symptoms, an observation that has practical implications for the future commercial development of X-2. Taken together, co-inoculation of X-2 with X-8 resulted in changes in the *S. japonica* transcriptome, up-regulating the genes related to the immune system and pathogen defense and down-regulating the gene involved in photosynthesis. This provides greater insight into the potential mechanisms by which X-2 protects its host from X-8-induced bleaching disease.

Beneficial microbes can alter host-derived metabolites to protect the host against multiple biotic threats (43, 44). Moreover, microorganism-derived metabolites also have capacity to assist plants to resist abiotic and biotic stresses (43, 44). It has been reported that beneficial microorganisms for corals can trigger metabolic alterations in coral holobionts, such as the degradation of dimethylsulfoniopropionate and the maintenance of lipids, which ultimately mitigated bleaching disease in corals (16). Our results also revealed significant metabolic differences between *S. japonica* holobiont when inoculated with either X-2 + X-8 or X-8 alone. For example, significantly higher levels of betaine were detected after 4 hours in the presence of X-2 compared to all other treatments. Betaine can act as an osmolyte in marine algae (45), and its increase might contribute to the resistance of *S. japonica* to pathogens. Interestingly, after 5 hours, the level of betaine showed the opposite patterns, further supporting the idea that X-2 mainly plays a protective role before the onset of bleaching disease. In addition, we observed significant correlations between the majority of DEGs, DMs, and *Vibrio* spp. after inoculation with X-2. In particular, *Vibrio* was classified as an indicator species in the X-2 + X-8 co-inoculated treatment. Therefore, the *S. japonica* epimicrobiota played a crucial role in the holobiont and the manipulation of the epimicrobiota was able to elicit changes in both the host microbiome and *S. japonica* to mitigate the occurrence of bleaching disease.

**Fig 5:**
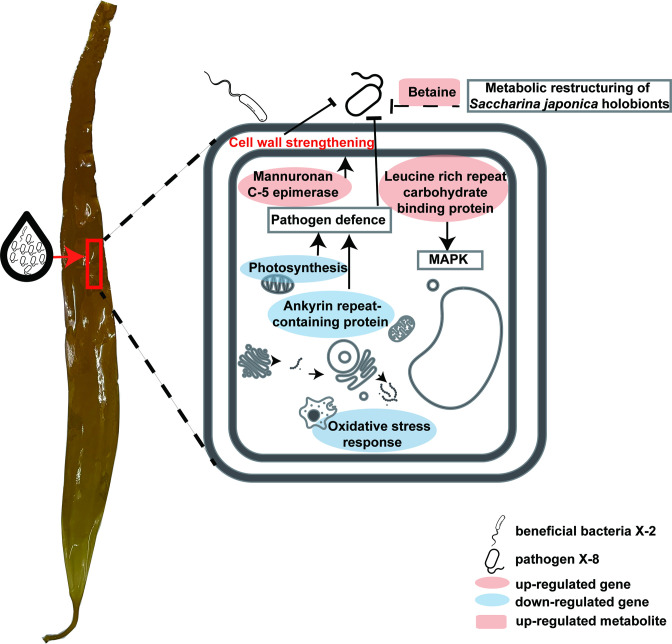
Schematic diagram of the differential response of the cellular components in *S. japonica* holobionts after inoculating with X-2 + X-8, compared to X-8 alone inoculated treatment. The icons of cellular components were obtained in thenounproject (https://thenounproject.com/).

Microbiome manipulation through X-2 triggers the restructuring of epibacterial communities and a suit of *S. japonica* responses, including the alteration of transcription (up-regulated genes involving in immune and stress protection pathways and down-regulated genes involving in photosynthetic energy synthesis) and metabolism (increased betaine abundance) (Fig. 5). These responses are believed to contribute to the overall enhancement of *S. japonica* resistance to pathogen-induced bleaching disease. Moreover, our results highlight that the application of beneficial bacteria before the onset of disease might be critical to disease outcome. This study supports the feasibility of microbiome manipulation through a beneficial bacterium to control disease in the farmed *S. japonica* and elucidates the underlying mechanisms by which it improves *S. japonica* health. Further studies should also focus on the application of beneficial bacteria in the cultivation of *S. japonica.*

## MATERIALS AND METHODS

### Experimental design

**Fig 6:**
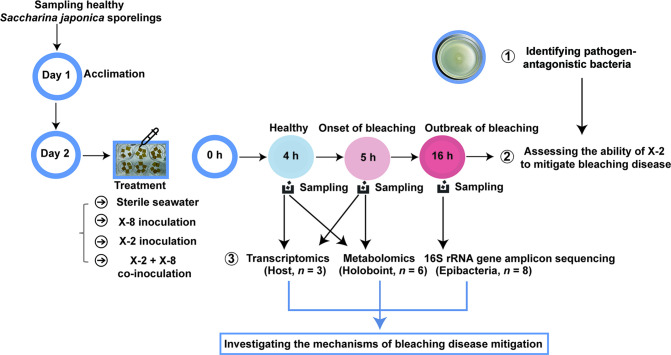
Schematic diagram of the experimental objectives and design in this study.

Schematic diagram of the objectives and design of this study is shown in Fig. 6.

### Confirmation, morphological characteristics, molecular identification, and phylogenetic analysis of a bacterium with the antagonistic activity against the causative agent of bleaching disease in *S. japonica*

X-2 was isolated simultaneously with X-8 from the same bleaching diseased sporelings (28). The Burkholder agar diffusion assay was used to assess the antagonistic activity of X-2 against the pathogen X-8 (46). Briefly, X-2 and X-8 were pre-cultured according to the method of Zhang et al. (28). Thereafter, the X-8 layer was prepared by mixing molten 0.6% (wt/vol) marine agar in seawater with 1% (vol/vol) suspension of the X-8 (optical density at 600 nm [OD_600_] ≈ 1.0). Then, 2 µL of X-2 (OD_600_ ≈ 1.0) was spotted onto the agar surface and incubated at 25°C (an antagonistic effect was indicated by the zone of growth inhibition with a diameter ≥ 7 mm). Experiments were conducted with three replicates.

Negative-staining transmission electron microscope (TEM) was used to observe the cell morphology of X-2. For negative-staining TEM, the X-2 suspension (OD_600_ ≈ 1.0) was pelleted by centrifugation (3,000× *g* for 10 minutes) and resuspended in 3.0% (wt/vol) glutaraldehyde for 10 minutes. The X-2 resuspension was then dropped onto a carbon-coated copper grid for 3 minutes, followed by drying at room temperature for 15 minutes. The sample was observed using JEM 1200EX (JEOL, Japan).

The full-length 16S rRNA gene sequence of X-2 was obtained from its whole genome sequences (National Center for Biotechnology Information [NCBI] BioProject: PRJNA810890 at https://www.ncbi.nlm.nih.gov/bioproject/PRJNA810890). The bacteria were then identified using the EzBioCloud server (http://www.ezbiocloud.net). To accurately identify the strain X-2, we also conducted the average nucleotide identity (ANI) analysis. Pairwise comparison of ANI between strain X-2 and its standard bacterial strain was analyzed based on BLAST+ (ANIb) using JSpeciesWSc (47). The two strains were regarded as the same species when the value of ANI ≥ 95%. Phylogenetic analysis of strain X-2 was further performed as follows: BLASTn analyses were performed against the 16S rRNA gene sequences database from the NCBI (https://www.ncbi.nlm.nih.gov/) using 16S rRNA gene sequence of the strain X-2 to select that of its closely related bacterial strains. All these sequences were conducted multiple sequence alignment using Clustal X (48). The maximum likelihood method with a 1,000 times bootstrap value was used to construct a phylogenetic tree by MEGA version X software (49).

### *S. japonica* infection assays

The putative beneficial bacterium X-2 was inoculated to *S. japonica* to assess its ability to mitigate bleaching disease. Healthy juvenile sporophytes (30–40 cm in length) were collected from Weihai Changqing Ocean Science & Technology Co., Ltd., Rongcheng, Shandong Province, China, in December 2021. Samples were transported to the laboratory, rinsed with sterile seawater at least three times to remove epiphytes, and incubated in a light incubator at 10 ± 0.5°C with a 12:12-hour light-dark photoperiod under 80 μE/m^2_·_^s for overnight acclimatization. Before infection experiments, the pre-cultured strains were centrifuged at 4,000× *g* for 20 minutes and then resuspended in sterile seawater. Healthy juvenile sporophytes were cut into small fragments (1.0 × 1.0 cm^2^) and placed into the individual wells of 6-well plates (Thermo Fisher Scientific, Inc., Waltham, MA, USA). To determine the optimum concentration ratio for the control of *S. japonica* bleaching disease by co-inoculation of X-2 + X-8, infection assays were performed in different concentration ratios of X-2: X-8 (1:1; 1:2; 1:4; 1:6; 1:8; 1:10; 1:12). Four treatments were conducted: (i) sterile seawater without inoculation of bacteria (control); (ii) X-8 inoculated treatment (10^8^ CFU/mL); (iii) X-2 inoculated treatment; and (iv) X-2 + X-8 co-inoculated treatment, X-8 with a final concentration of 10^8^ CFU/mL. Each treatment was performed with 30 biological replicates. The 24-well plates were incubated in a light incubator at 10 ± 0.5°C with 12:12 hour light–dark photoperiod under 80 μE/m^2_·_^s, followed by sampling in 2-hour intervals within 0–24 hours for microscopic observation using a light microscope (Nikon, Japan) and comparing the proportion of the bleached *S. japonica* replicates, respectively. Statistical analyses were conducted using SPSS 22.0 (IBM, Inc, Armonk, NY, USA). All data were analyzed using one-way analysis of variance (one-way ANOVA) and Tukey’s tests with *P* < 0.05 indicated statistically significant differences.

### *S. japonica* epibacterial community analysis via 16S rRNA gene amplicon sequencing

The epibacterial community of *S. japonica* was assessed by 16S rRNA gene amplicon sequencing. According to our previous study (29), the colonization of X-8 may take longer. The *S. japonica* samples were collected at 16 hours (proportion of bleached individuals significantly increased) after exposure to control (sterile seawater), X-2 + X-8 co-inoculated, X-2 inoculated, and X-8 inoculated treatments, and subsequently rinsed with sterile seawater to remove the loosely attached bacteria. Each treatment was performed with eight biological replicates. The epibacterial communities of *S. japonica* (50 cm^2^) were swabbed using sterile cotton swabs and then stored at –80°C. Microbial DNA was extracted using the HiPure Soil DNA Kits (Magen, Guangzhou, China) according to the manufacturer’s instructions. DNA concentrations were measured using NanoDrop ND-1000 (Thermo Fisher Scientific, Inc., Waltham, MA, USA), and the quality was assessed by agarose gel electrophoresis on a 1.0% agarose gel. The V3–V4 regions of the 16S rRNA genes were amplified using primers 341F: CCTACGGGNGGCWGCAG; 806R: GGACTACHVGGGTATCTAAT with the following procedures: 95°C for 2 minutes, 27 cycles at 98°C for 10 seconds, 62°C for 30 seconds, and 68°C for 30 seconds, and a final extension at 68°C for 10 minutes. Furthermore, the PCR products were separated on 1.0% agarose gels and purified using the AxyPrep DNA Gel Extraction Kit (Axygen Biosciences, USA). They were then quantified using the ABI StepOnePlus Real-Time PCR System (Life Technologies, Foster City, CA, USA). The purified PCR products were added with multiplexing indices and Illumina sequencing adapters and then pooled in equimolar and paired-end sequenced (PE250) on the Illumina Novaseq6000 platform by Guangzhou Genedenovo Biotechnology (Guangzhou, China).

Raw reads containing primer sequences and unknown nucleotides were filtered and truncated to obtain clean reads. Dereplication and denoising were then performed using DADA2. The paired-end denoised reads, with a minimum of 12-bp overlap were merged as raw amplicon sequence variants (ASVs). Chimera sequences were identified and deleted using UCHIME algorithm (50) to obtain the chimera-free ASV sequences. Chimera-free ASV sequences and their abundances were then exported. The tag sequence with the highest abundance was selected as the representative sequence within each cluster. The representative ASV sequences were classified into organisms by a naive Bayesian model using the RDP classifier (51) based on the SILVA database (https://www.arb-silva.de/) (52), with a confidence threshold of 0.8. Sequences classified as chloroplast, mitochondria, and archaea origin were removed from the data set.

To confirm the presence of the inoculated bacteria (X-2 and X-8) in the host epibacterial community, 16S rRNA gene sequences of these bacteria were searched against the representative sequences of ASVs from different treatments using BLAST 2.2.30+ (53). ASV sequences with 100% identity were regarded as to be corresponding to X-2 and X-8, respectively. These ASVs’ comparisons among different treatments were calculated using Tukey’s honestly significant difference (HSD) test in the R project Vegan package (version 2.5.3). Subsequent bacterial community analyses were conducted after subtracting the X-2 and X-8 reads from the data set. The stacked bar plot of the community composition was visualized using R project ggplot2 package (version2.2.1). To determine the genera associated with the community differences, genera with relative abundance ≥ 1% were performed IndVal calculation and statistical test using the R project labdsv package. Genera with significantly higher IndVal (*P* < 0.05) were considered as the indicator species. Alpha diversity analyses, including Chao1, Shannon index, and Good’s coverage, were performed using QIIME (54) (version 1.9.1). Comparison of alpha diversity between different treatments was calculated by Tukey’s HSD test using the R project Vegan package (version 2.5.3). The difference in bacterial community structure (beta diversity) was analyzed based on Bray–Curtis dissimilarity. Non-metric multi-dimensional scaling (NMDS) was generated using the R project Vegan package (version 2.5.3) and plotted using the R project ggplot2 package (version 2.2.1). Permutational multivariate analysis of variance (PERMANOVA) was conducted to evaluate the differences between the different treatments using the R project Vegan package (version 2.5.3), while permutational analysis of multivariate dispersions (PERMDISP) was conducted to evaluate the dispersions of the epibacterial community structure between the different inoculated treatments using the R project Vegan package (version 2.5.3).

### *S. japonica* transcriptomic analysis

The *S. japonica* gene expression responses to the protective bacterium X-2 were investigated by RNA-sequencing (RNA-seq). The *S. japonica* samples were collected after 4 hours (before the onset of bleaching symptom) and 5 hours (the onset of bleaching symptom) of exposure to control, X-2 + X-8 co-inoculated, X-2 inoculated, and X-8 inoculated treatments, and subsequently rinsed with sterile seawater to remove the loosely attached bacteria. Each treatment was performed with three biological replicates. Tissue samples were frozen in liquid nitrogen and stored at −80°C. Total RNA was extracted from each sample (100 mg) using Trizol reagent kit (Invitrogen, Carlsbad, CA, USA) according to the manufacturer’s protocol. RNA quality was determined using an Agilent 2100 Bioanalyzer (Agilent Technologies, Palo Alto, CA, USA) and RNase-free agarose gel electrophoresis. The mRNA was then enriched using Oligo (dT) beads. Then the enriched mRNA was reverse-transcribed into cDNA with random primers and synthesized second-strand cDNA. The cDNA fragments were further purified with QiaQuick PCR extraction kit (Qiagen, Venlo, The Netherlands), end repaired, poly(A) added, and ligated to Illumina sequencing adapters. The ligation products were size selected by agarose gel electrophoresis, PCR amplified, and paired-end sequenced using Illumina HiSeq2500 by Gene Denovo Biotechnology Co. (Guangzhou, China). Raw reads were filtered using fastp (55) (version 0.18.0) to remove reads containing adapters or more than 10% of unknown nucleotides and low-quality reads containing more than 50% of low-quality (*Q* value ≤ 20) bases. Reads were then mapped to the rRNA database using short-reads alignment tool Bowtie2 (56) (version 2.2.8). The rRNA-mapped reads were removed to ensure that there was no rRNA contamination. The remaining clean reads were used in the subsequent assembly and gene abundance calculations. An index of the *S. japonica* reference genome (https://db.cngb.org/search/project/CNP0000364/) was built, and paired-end clean reads were mapped to the reference genome using HISAT2. 2.4 (57) with default settings. The mapped reads were assembled using StringTie v1.3.1 (58). Fragment per kilobase of transcript per million mapped reads (FPKM) value was calculated to quantify the expression level of each transcript. Gene function annotation was based on the following databases: NCBI non-redundant protein sequences (Nr), NCBI non-redundant nucleotide sequences (Nt), Protein family (Pfam), manually annotated and reviewed protein sequence database (Swiss-Prot), KEGG Ortholog database (KO), and Gene Ontology (GO). Principal component analysis (PCA) based on gene expression levels was performed using the R package gmodels. PERMANOVA was conducted to evaluate the differences between different treatments using the R project Vegan package. Differential expression analysis was conducted using DESeq2 (59) and genes with *P* < 0.05 and fold change (FC) > 2 or < 0.5 were considered as differentially expressed genes (DEGs). Pathway enrichment analysis of the DEGs was conducted by mapping to the KEGG pathway database (60).

To validate the RNA-seq data, we selected five DEGs in different pathways to conduct qRT-PCR. RNA obtained in RNA-seq was reverse-transcribed to cDNA using HiScript II First Strand cDNA Synthesis Kit (Vazyme, Nanjing, China) following the manufacturer’s instructions. The qRT-PCR assays were performed on a Roche LightCycler 480 system using THUNDERBIRD SYBR qPCR Mix (TOYOBO, Japan). The gene *actin* was used as the reference gene for internal control. The qRT-PCR program was set as follows: 95°C for 30 seconds, 40 cycles at 95°C for 15 seconds, 60°C for 30 seconds, and 72°C for 30 seconds. Each reaction was performed in three biological replicates. The relative expression level of each gene was calculated using the 2^-∆∆*Ct*^ method. Least significant difference (LSD) tests with *P* < 0.05 indicated statistically significant differences. Primer information is exhibited in Table S10.

### *S. japonica* holobionts’ metabolomic analysis

The holobionts of *S. japonica* metabolic responses to the beneficial bacterium X-2 were investigated by metabolomics. The *S. japonica* samples were collected after 4 hours (before the onset of bleaching symptom) and 5 hours (the onset of bleaching symptom) of exposure to control, X-2 + X-8 co-inoculated, X-2 inoculated and X-8 inoculated treatments, and subsequently rinsed with sterile seawater to remove the loosely attached bacteria. Each treatment was performed with six replicates. The tissues samples were frozen in liquid nitrogen and then stored at −80°C. Each 100 mg sample was grounded into a fine powder, then 1,000 µL methanol–acetonitrile–H_2_O (2:2:1, vol/vol/vol) was added to the homogenized solution for metabolite extraction. The mixture was centrifuged at 14,000× *g* for 15 minutes at 4°C. After that, the supernatant was dried in a vacuum centrifuge. The samples were re-dissolved in 100 µL acetonitrile–water (1:1, vol/vol) solvent for liquid chromatography–mass spectrometry (LC-MS) analysis. Quality control (QC) samples were prepared by pooling 10 µL of each sample and analyzed together with the other samples to monitor the stability and repeatability of instrument analysis. The LC-MS analysis was conducted using an Ultra Performance Liquid Chromatography and High-Resolution Mass Spectrometry (UHPLC) (1290 Infinity LC, Agilent Technologies) coupled with a quadrupole time-of-flight (AB Sciex TripleTOF 6600) in Shanghai Applied Protein Technology Co., Ltd.

Samples were analyzed using a 2.1 mm × 100 mm ACQUIY UPLC BEH 1.7-µm column (Water, Ireland) for hydrophilic interaction liquid chromatography (HILIC) separation. We used both electron spray ionization (ESI) positive (ESI+) and negative (ESI−) modes. In these two modes, the mobile phase A containing 25 mM ammonium acetate and 25 mM ammonium hydroxide in water and B containing acetonitrile. The gradient elution procedure is as follows: 85% B for 1 minute, linearly reduced to 65% in 11 minutes, reduced to 40% in 0.1 minute and kept for 4 minutes, increased to 85% in 0.1 minute, with a 5-minute re-equilibration period employed. After HILIC separation, the ESI source conditions were set as follows: Ion Source Gas1 (Gas1) as 60, Ion Source Gas2 (Gas2) as 60, curtain gas (CUR) as 30, source temperature: 600°C, IonSpray Voltage Floating ± 5,500 V. In MS-only acquisition, the instrument was set to acquire over the *m*/z range 60–1,000 Da, and the accumulation time for TOF MS scan was set at 0.20 seconds/spectra. In auto-MS/MS acquisition, the instrument was set to acquire over the *m*/z range 25–1,000 Da, and the accumulation time for product ion scan was set at 0.05 seconds/spectra. The product ion scan is acquired based on the information-dependent acquisition with high sensitivity mode. The parameters were set as follows: the collision energy was fixed at 35 V with ± 15 eV; declustering potential, 60 V (+) and −60 V (−); exclude isotopes within 4 Da; and candidate ions to monitor per cycle: 10.

The raw data files generated by UHPLC-MS/MS were processed using the Compound Discoverer 3.1 (CD3.1, Thermo Fisher) to perform peak alignment, peak picking, and quantitation for each metabolite. Peak intensities were then normalized to the total spectral intensity. The normalized data were used to predict the molecular formula based on additive ions, molecular ion peaks, and fragment ions. Then peaks were matched with the mzCloud (https://www.mzcloud.org/), mzVault, and Masslist databases to obtain accurate qualitative and relative quantitative results. Multivariate analyses, including partial least squares discriminant analysis (PLS-DA) and orthogonal projection to latent structures-discriminant analysis, were performed using R project ropls package. Metabolites with variable important in projection (VIP) > 1, *P* (*t*-test) < 0.05, and FC > 1.5 or < 0.667 were considered as differential metabolites (DMs). Pathway enrichment analyses of the DEGs were conducted by mapping to the KEGG pathway database.

### Epibacterial community, transcriptome, and metabolome correlation analyses

Correlation analyses between top 20 ASVs, all DMs with annotation, and main DEGs were conducted using Spearman correlation analysis. Spearman correlation coefficient and *P*-value were calculated using R function, cor. and cor. test, with *P* < 0.05 indicated significant correlation. Correlation heatmap was generated using R corrplot package.

### Supplemental material

The supplementary material for this article can be found online at https://doi.org/10.6084/m9.figshare.22337065.v4

## Data Availability

All data are available in the main text or the supplementary materials. The raw reads of 16S rRNA gene amplicon sequencing were deposited into the NCBI Sequence Read Archive (SRA) database (accession no. PRJNA874856). The raw reads of transcriptomic sequencing were deposited into the NCBI SRA database (accession no. PRJNA875568).
